# Premiums on Child-Murder

**Published:** 1889-02-23

**Authors:** 


### The Hospital

A Weekly Institutional Journal of

**Science, Medicine, Nursing, and Philanthropy.**

*For Hospitals, Asylums, Medical Practitioners, Students, Nurses, Families, Parishes, Congregations, and the Charitable Public.*

Vol. V. No. 126.] [February 23, 1889.

### Premiums on Child-Murder.

"The poor in a loomp is baad," said the "Northern Farmer." We do not believe it. There is in every class, not excepting the very highest, a residuum of rascality. But the poor, being the most numerous class, produces the largest number of black sheep; and their blackness, being of a coarser kind, is apt to look more black than that of their more polished, but not whiter, fellow-scoundrels. If the receiver be as bad as the thief, the tempter must be held as guilty as the tempted, if, indeed, not more guilty. Few men, if asked to judge between Mephistopheles and Faust, would hesitate in awarding the prize of demerit. The tempter takes the palm for wickedness, although the tempted who falls be adjudged a partaker of his guilt.

A system of life assurance has gradually grown up in this country which results in that most horrible of all crimes — deliberate child-murder. Such crime brands with infamy not only the monsters who commit it, and the tempters who make it profitable for them to do so, but also the community who, seeing it done in their midst, make no sufficient effort to stamp it out at once and for ever. Such crime can be partly cured by legislation, and by no other means which lie immediately to hand. The legislation required for a purpose of this kind would be comparatively simple; and what is of equal importance, would be practically non-controversial. It must proceed on lines which make child-murder unprofitable to those who commit it. That is the very core of the matter. The class of poor persons whose dispositions are selfish and criminal will do murder if it pays them to do it, but will avoid it if the profit lies all the other way. It may be dreadful to have to admit facts like these, but it is much more dreadful to admit them and to take no steps to render them impossible. The community, we ourselves, share the guilt of the "murder of the innocents," every moment we waste in mere idle bemoaning of the subject. It is well within the power of the Legislature to frame and pass such measures of restriction as shall at once remove all temptations from parents and guardians to take away the lives of their children for the sake of "the insurance money."

Many of the oldest and most reputable assurance offices do not insure young children at all. The business is confined to a few offices which make more or less of a specialty of it. An important fact bearing upon the whole question is that the offices which assure children's lives "make it pay." It is clear, therefore, that only a small percentage of those actually assured die in childhood; for if the percentage were relatively large, the business, which is done at low premium rates, and which costs probably not less than fifty per cent. on an average in management expenses alone, would not pay, and consequently would not be done. This fact throws an important light on the whole subject; for it shows beyond a doubt that, notwithstanding the loud outcry which has been made, the actual proportion of child-deaths is relatively small, whilst the proportion of child-murders must be infinitely smaller still. That is a reason for taking a calm and dispassionate view of the subject; but it is no reason at all for postponing consideration of it altogether. So long as the law permits assurance societies to make the deaths of children profitable to other persons than the children, so long must those of us who are responsible for the state of the law be held to have a certain complicity in the crime of child murder.

We have the authority of men of the first rank in the insurance world for the statement that there is no just ground for objecting to the assurance of children's lives. Not only so, but such assurance can be made of great practical advantage to honest and industrious people. To many of the poor a doctor's bill and a funeral may be the last pound added to the load which finally sinks them into the mire of pauperism and despair. They take out policies on their children's lives in order that in the event of death they may have the wherewithal to bury them respectably, and to settle the doctor's claims. This, in itself, is not only an innocent, but even a commendable proceeding. No such change in the law should be made as will interfere with the effecting of a provision of this kind. What is wanted is—not the abolition, but the regulation of child insurance. Mr. Braxton Hicks, speaking from his experience as a metropolitan coroner, draws a black picture of the many different lines which converge towards child-murder, and of the fiends of all sorts who travel on those lines. There can be no doubt that baby farmers, mothers of illegitimate children, brutal and selfish parents, who find a family burdensome—these and many others first get their charges accepted by an insurance office, and then set themselves by methods which are quick or slow, savagely or deliberately murderous, to take away the lives of the unfortunate and helpless creatures whom nature and humanity would prompt them to protect. Merely to think of it is to excite an indignation which would sweep away at a single blow every assurance office which lends itself to such horrors. But that would be neither just to the offices nor kind to the poor. Regulation by statute and not abolition is the peremptory need of the moment.

The evil fact upon which Mr. Braxton Hicks insists, and which is recognised by some of the most eminent assurance authorities who do not compete for children's assurances, is that in the event of an insured child's death the responsible guardian reaps a monetary advantage. This is the bait which is constantly dangled before the eyes of poor, greedy, and brutal wretches by the agents of certain assurance companies. The bait may appear to many people so small as to be utterly powerless to persuade either man or woman to crime. But it is found in practice that guardians of poor children, and even their own parents, will not only look at it, but look and look again, until they will commit foul and deliberate murder, even child-murder, in order to make it their own. All this must be put an end to. "How oft the sight of means to do ill deeds makes ill deeds done!" Never was truer word said by Shakespeare or anyone else. Then let the means we have been heie discussing, as well as the sight of them, be rendered impossible. If what is wanted be a decent funeral for a dead child and the payment of the doctor's bill, what is to prevent assurance companies themselves providing for the funeral and paying the doctor too? Perhaps the mere fact of the company's providing and paying the doctor would be the most efficient check which could be put upon child-murder. It is a company's interest to keep assured children alive. They thereby of course save their own funds. Let them then contract to pay for medical attendance as well as for the funeral, and let such attendance be under their own immediate supervision. Both doctor and company will thus have a direct interest in keeping sickly children alive, and it will be very strange if with such a check as the daily visit of a doctor, to whom a live patient is more profitable than a dead one, any untutured murderer shall find it possible to carry out his diabolical purpose. At present, so far as is known, insured children are left absolutely at the mercy of those who have a direct pecuniary interest in their death.

It is not unlikely that there has been some exaggeration as to the number of helpless children who have been foully killed for the sake of the few pounds which an assurance office had contracted to pay. But that is of no moment. What every right-minded man and woman feels is that it should be made absolutely impossible for even one child to be murdered with the sole object of profit to the murderer. If the death of an assured child be made unprofitable to those who have charge of him, and if the care of his health be given into the hands of an assuring office which in addition to the direct interest it has in the preservation of his life, is charged by statute with the responsibility of supplying medical attendance, a safeguard will be placed around helpless children of the greatest practical value. Not until some efficient checks of this kind are made compulsory can the conscience of the nation be in any degree satisfied.

